# Editorial: From “Junk DNA” to Clinically Relevant Tools for Cancer Diagnosis, Staging, and Tailored Therapies: The Incredible Case of Non-Coding RNAs

**DOI:** 10.3389/fonc.2019.00389

**Published:** 2019-05-14

**Authors:** Marilena V. Iorio, Dario Palmieri

**Affiliations:** ^1^Molecular Targeting Unit, Experimental Oncology Department, Fondazione IRCCS Istituto Nazionale dei Tumori, Milan, Italy; ^2^IFOM (Fondazione FIRC Oncologia Molecolare), Milan, Italy; ^3^Department of Cancer Biology and Genetics, The Ohio State University, Columbus, OH, United States

**Keywords:** ncRNA, microRNA, circRNA, resistance, cancer diagnosis and intervention

In recent years, a growing body of evidence indicates that cell genetic behavior and fate is not merely driven by the limited number (~21,000) of protein-coding genes and their regulatory regions. Conversely, non-coding genomic regions, originally labeled as “junk DNA,” have been demonstrated to be transcriptionally active (although not translated into proteins) and to play causal roles in cell physiology and pathology.

It is now well-known that these non-coding RNAs (ncRNAs) are not transcriptional noise, but they are critically involved in a number of processes such as differentiation, development, inflammation, immune response, and cancer. Their biological relevance has been confirmed by comparative genome studies demonstrating that species degree of complexity correlates with the number of non-coding genes more than protein-coding genes.

However, the biological mechanisms through which ncRNAs exert their functions have only been partially elucidated. This is especially the case of the about 9,000 small (under 200 bp) ncRNAs, and particularly microRNAs, which inhibit gene expression at post-transcriptional level by preventing translation of complementary mRNA by binding to their 3'UnTranslated Regions (UTRs). On the other hand, the long (200 bp−100 kb) ncRNAs family, encompassing more than 40,000 members, still requires extensive effort to obtain a comprehensive understanding of their molecular details and mechanisms of action.

As far as microRNAs are concerned, several groups have demonstrated their causal role in cancer pathogenesis, and extensive studies have attempted to modulate the small non-coding transcriptome (by abrogating or recovering the expression of oncogenic and tumor-suppressive miRNAs, respectively) as a therapeutic approach for cancer. Unfortunately, the promise of microRNA-based anti-cancer drugs is still far from the clinical use, especially due to the lack of appropriate cancer-specific delivery systems. Conversely, the accurate quantification of microRNAs from cancer patients, both in neoplastic lesions and in liquid biopsies, might represent an invaluable tool for tumor classification, staging, and to provide patients with the most appropriate clinical care.

In this Research Topic, Laprovitera et al. provide a method for the accurate and absolute assessment of multiple microRNA levels in paraffin-embedded tissues using EvaGreen-based droplet digital PCR technology, potentially applicable also to miRNAs circulating in biological fluids and in multiple subcellular compartments such as exosomes and microvesicles. This approach would allow the discovery and the validation of miRNAs as biomarkers in a number of different tumor types (Laprovitera et al.). In fact, to date, one of the drawbacks of the comparison of multiple studies for the validation of microRNAs as biomarkers is represented by the absence of a standardized and absolute quantification method. This concern is specifically raised by Lo Russo et al. while analyzing the potential clinical impact of microRNA quantification in tumors, blood samples and pleural effusions of Malignant Pleural Mesothelioma (MPM) patients. In an extensive literature analysis, the authors describe the great potential of miRNAs from both a diagnostic and therapeutic point of view. However, because of the heterogeneity of the analyzed studies, the authors urge a coordinated collaboration among research and clinical groups to implement miRNA-based diagnostic/prognostic systems in the clinical settings (Lo Russo et al.).

Despite the development of several new therapeutic protocols and targeted drugs, including an ever-growing number of small molecules with enhanced efficacy in the treatment of cancer patients of different type, resistance still represents an extremely frequent phenomenon. When resistance to treatments arises, cancer patients switch to different (and potentially less effective or more toxic) approaches until they run out of options. For these reasons, it is crucial that clinicians can identify those patients who could benefit of specific drugs, sparing them unnecessary toxicities and providing them with the best possible clinical treatment. Non-coding RNAs represent valid biomarkers predictive of response to several different conventional and new-generation anti-neoplastic treatments. Plantamura et al. specifically reviewed the relevance of microRNAs as modulators of the cellular DNA damage response (DDR) by targeting DNA-repair genes such as ATM, BRCA1/2, and DNA-PK. Since many chemo- and radio-therapeutic agents act by inducing damages to the genomic DNA, a dysfunctional DDR (dependent on microRNA-dysregulation) could lead to potential sensitivity/resistance to these treatments (Plantamura et al.).

In the review from Hahne and Valeri an extensive analysis of non-coding RNAs involved in the resistance to anti-cancer drugs in gastrointestinal tumors was performed. The review reports evidence of the role of both microRNAs and long non-coding RNAs as central hubs for the development of drug resistance mechanisms, including those related to DDR. However, as indicated by the authors, the current potential clinical relevance for microRNAs and other non-coding RNAs is represented by their role as tissues or biofluids biomarkers that could potentially show, in a cost-effective way, their utility to monitor patient response or forecast treatment resistance (Hahne and Valeri).

The role of non-coding RNAs in cancer drug resistance and the extensive network of interactions between ncRNAs and anti-neoplastic therapies are also the subject of the review from Corrá et al. This study contains an extremely interesting network analysis, focused on miRNAs and long non-coding RNAs playing the most central role in chemoresistance. Notably, the authors also focused on ncRNAs associated to a limited number of drugs, and generated a clustering analysis that could potentially help in identifying the cross-talk between non-coding RNAs and multiple treatment options (Corrá et al.).

Dragomir and Calin provided an extensive review of a novel and extremely interesting class of non-coding RNAs, called circular RNAs (circRNAs). A limited number of biological functions for this class of RNAs have been clearly described so far, including their ability to “sponge” microRNAs and preventing their ability to modulate gene expression. Noteworthy, the authors describe how this ncRNA family could be either common driving mechanism of oncogenesis, or common byproduct/end-products. For these reasons, they suggest to treat circRNAs, especially those found in serum/plasma, with caution, especially based on the experience obtained in the last years with microRNAs, until a better understanding of their biogenesis, secretion, and molecular roles is gained. This approach will prevent supplementary errors and data misinterpretation to be considered before their use as cancer biomarkers (Dragomir and Calin). In line with the growing interest toward circRNAs, an original research article from Shao et al. showed that specific circular RNAs where differentially expressed in pancreatic ductal adenocarcinoma (PDAC) cell lines and in the plasma of PDAC patients, suggesting a potential role as predictive biomarkers with a causal role in the sensitivity to gemcitabine.

In summary, this Research Topic covered multiple basic, technical, and clinical issues regarding the multiple classes of ncRNAs and highlights their impact both in our understanding of cancer biology and in their relevance as biomarkers predictive of clinical outcome or response to therapies.

## Author Contributions

MI and DP wrote and edit the manuscript.

### Conflict of Interest Statement

The authors declare that the research was conducted in the absence of any commercial or financial relationships that could be construed as a potential conflict of interest.

